# Effect of Blood Transfusion on Short- and Long-Term Outcomes in Oral Squamous Cell Carcinoma Patients Undergoing Free Flap Reconstruction

**DOI:** 10.3389/fsurg.2021.666768

**Published:** 2021-05-18

**Authors:** Aimin Feng, Jiaqiang Zhang, Xihua Lu, Qigen Fang

**Affiliations:** ^1^Department of Anesthesiology and Perioperative Medicine, Henan Provincial People's Hospital, People's Hospital of Zhengzhou University, Zhengzhou, China; ^2^Department of Anesthesiology and Perioperative Medicine, Henan Cancer Hospital, The Affiliated Cancer Hospital of Zhengzhou University, Zhengzhou, China; ^3^Department of Head and Neck Thyroid, Henan Cancer Hospital, The Affiliated Cancer Hospital of Zhengzhou University, Zhengzhou, China

**Keywords:** perioperative blood transfusion, oral squamous cell carcinoma, free flap reconstruction, survival analysis, complication

## Abstract

**Purpose:** To analyze the short- and long-term effect of perioperative blood transfusion (PBT) in patients undergoing surgical treatment for oral squamous cell carcinoma (SCC).

**Methods:** Patients undergoing free flap reconstruction were retrospectively enrolled and divided into two groups based on the implementation of PBT. Flap revision, surgical site infection (SSI), flap failure, overall survival (OS), and disease-specific survival (DSS) were compared between the two groups.

**Results:** In 170 patients with PBT, 10 (5.9%) flaps required exploration revision, SSI occurred in 18 (10.6%) patients, and flap necrosis was noted in 6 (3.5%) patients. These rates were comparable to those in patients without PBT. The two groups had similar DSS rates, but the 5-year OS rates were 49 and 59% in patients with PBT and without PBT, respectively. This difference was significant. Patients with 4 units of PBT had OS rates comparable to those of patients with >4 units of PBT. A Cox model confirmed the fact that the decrease in OS was independent of PBT.

**Conclusion:** In patients with free flap reconstruction for oral SCC, PBT did not increase the short-term complication rate or cancer-linked mortality. However, it was related to an elevated overall risk of death.

## Introduction

Oral squamous cell carcinoma (SCC) is the most common malignancy in cancers of the head and neck (1), usually presenting as advanced-stage disease on initial treatment. Due to its site specificity, advanced-stage patients sometimes complain of poor appetite, pain, and dysphagia (2), and a number of patients have anemia with different degrees of severity. Additionally, in these patients, a free flap reconstruction is frequently required, and intraoperative blood loss is significant (3). Therefore, perioperative blood transfusion (PBT) is not uncommon in patients receiving free flap reconstruction for oral SCC.

Immunosuppression induced by PBT was first discovered by Opelz et al. (4) in a renal transplant. A series of studies thereafter have proven the adverse influence of PBT on oncologic survival in cholangiocarcinoma (5) and liver (6), bladder (7), pancreatic (8), gastric (9), and lung cancer (10). However, the effect on oral SCC remains controversial. A summary by Szakmany et al. (11) reported that some studies reported no significant effect of PBT, while some noted a decreased survival rate related to PBT in a dose-dependent manner. Therefore, in the current study, we aimed to analyze the short- and long-term effect of PBT in patients undergoing free flap reconstruction for oral SCC.

## Methods

### Ethical Consideration

The institutional research committee of Zhengzhou University approved our study, and all participants provided written informed consent for medical research prior to the initial treatment. All experiments were performed in accordance with the relevant guidelines and regulations.

From January 2010 to December 2018, all patients undergoing free flap reconstruction for untreated primary oral SCC were retrospectively enrolled. Thereafter, data regarding demography, systemic disease, including hypertension, diabetes and cardiovascular and cerebrovascular diseases, TNM stage based on the 8th AJCC classification, PBT, ASA status, Eastern Cooperative Oncology Group (ECOG) status, preoperative serum albumin level, postoperative pathology, treatment, and follow-up were acquired and analyzed.

The perioperative period was defined as the timespan between the start of the patient's hospitalization and the time they left the surgery department. All patients underwent systemic preoperative examinations, including ultrasound, CT, MRI and/or PET-CT imaging. Pathological sections were reviewed by at least two head and neck pathologists. Perineural invasion (PNI) was considered present if tumor cells were identified within the perineural space and/or nerve bundle. Lymphovascular infiltration (LVI) was considered positive if tumor cells were noted within the lymphovascular channels (12–16). The depth of invasion was measured from the level of the adjacent normal mucosa to the deepest point of tumor infiltration, regardless of the presence or absence of ulceration (12). The indications for adjuvant treatment included neck lymph node metastasis, PNI, LVI, high tumor stage, positive margins, and extracapsular spread (12). Following discharge, patients were followed every 3 months during the first year, every 6 months during the second year, and once a year after the second year (17).

In general, our blood units used were all leukocyte-depleted, and a preoperative hemoglobin level of <60 g/L required to increase it to at least 80 g/L. PBT was selectively performed under the consideration of the surgeon and the anesthesiologist if the hemoglobin level was 60–90 g/L. The choice to undertake an intraoperative blood transfusion depended on the preoperative hemoglobin level, intraoperative blood loss, and the patient's general condition. Postoperative blood transfusion was performed mainly based on the latest hemoglobin level.

All patients were managed according to the same method. The postoperative flap was observed hourly during the first 24 h and then every 4 h for the next 3 days. If there was a suspicion of vascular events, an exploration operation was immediately performed. All patients received prophylactic anti-infective therapy with cephalosporin 30 min prior to incision, which was continued for at least 3–5 days following surgery. Nasogastric nutrition was routinely administered until patients could eat by mouth.

Short-term outcomes included flap revision, surgical site infection (SSI), and flap necrosis. Flap revision was defined as a surgical intervention complicated by a postoperative vascular event. SSI was defined as wound dehiscence with salivary leak or purulent drainage (18), and flap necrosis was defined as total flap failure. The association between short-term outcomes and PBT was analyzed using a Chi-square test.

Long-term outcomes included overall survival (OS) and disease-specific survival (DSS). The survival time of OS was calculated from the date of operation to the date of death from any cause or the last follow-up. Similarly, the survival time of DSS was calculated from the date of operation to the date of cancer-caused death or the last follow-up. The association between long-term outcomes and PBT was analyzed using a Kaplan-Meier method and Cox model.

All statistical analyses were performed with SPSS 20.0, and a value of *p* < 0.05 was considered to be significant.

## Results

A total of 423 patients were enrolled, with a mean age of 55.4 years. There were 324 (76.6%) males and 99 (23.4%) females and 288 (68.1%) smokers and 187 (44.2%) drinkers. Systemic disease was noted in 269 (63.6%) patients. ASA status was I in 154 (36.4%) patients, II in 191 (45.2%) patients, and III in 78 (18.4%) patients. ECOG status was 0 in 239 (56.5%) patients, 1 in 149 (35.2%) patients, and 2 in 35 (8.3%) patients. A total of 323 (76.4%) and 100 (23.6%) patients had normal and low preoperative serum albumin levels, respectively. Primary cancer sites were in the tongue in 171 (40.4%) patients, the mouth in 107 (25.3%) patients, the floor of the mouth in 85 (20.1%) patients, and the lower gingiva in 60 (14.2%) patients. A total of 100 (23.6%) patients had a pathologic T2 tumor, 234 (55.3%) patients had a pathologic T3 tumor, and 89 (21.0%) patients had a pathologic T4 tumor. Tumor differentiation was good in 126 (29.8%), moderate in 211 (49.9%), and poor in 86 (20.3%). Positive margin occurred in 63 (14.9%) patients. All patients underwent a neck dissection, and pathologic neck metastasis was confirmed in 221 (52.2%) patients. Patients with PBT had a poorer ECOG status (*p* = 0.040), more systemic disease (*p* = 0.004), and a poorer ASA status (*p* = 0.003) than patients without PBT. The two groups had no significant difference regarding other clinical and pathologic data (all *p* > 0.05) (Table 1).

**Table 1:** Demographic and pathologic information in the 423 enrolled patients.

**Variables**	**Number (*n* = 423,%)**	PBT^*^ (*n* = 170,%)	**No PBT (*n* = 253,%)**	***p***
Age				
<40 years	35 (8.3%)	15 (8.8%)	20 (7.9%)	
≥40 years	388 (91.7%)	155 (91.2%)	233 (92.1%)	0.737
Sex				
Male	324 (76.6%)	133 (78.2%)	191 (75.5%)	
Female	99 (23.4%)	37 (21.8%)	62 (24.5%)	0.514
Systemic disease	269 (63.6%)	122 (71.8%)	147 (58.1%)	0.004
Smoker	288 (68.1%)	119 (70.0%)	169 (66.8%)	0.489
Drinker	187 (44.2%)	68 (40.0%)	119 (47.0%)	0.153
ASA status				
I	154 (36.4%)	48 (28.2%)	106 (41.9%)	
II	191 (45.2%)	80 (47.1%)	111 (43.9%)	
III	78 (18.4%)	42 (24.7%)	36 (14.2%)	0.003
ECOG				
0	239 (56.5%)	86 (50.6%)	153 (60.5%)	
1	149 (35.2%)	64 (37.7%)	85 (33.6%)	
2	35 (8.3%)	20 (11.8%)	15 (5.9%)	0.040
Preoperative serum albumin level				
Normal	323 (76.4%)	126 (74.1%)	197 (77.9%)	
Low	100 (23.6%)	44 (25.9%)	56 (22.1%)	0.374
Primary cancer site				
Tongue	171 (40.4%)	71 (41.8%)	100 (39.5%)	
Buccal	107 (25.3%)	45 (26.5%)	62 (24.5%)	
The floor of the mouth	85 (20.1%)	30 (17.6%)	55 (21.7%)	
Lower gingiva	60 (14.2%)	24 (14.1%)	36 (14.2%)	0.770
Pathologic tumor stage				
T2	100 (23.6%)	41 (24.1%)	59 (23.3%)	
T3	234 (55.3%)	101 (59.4%)	133 (52.6%)	
T4	89 (21.0%)	28 (16.5%)	61 (24.1%)	0.157
Pathologic nodal status				
N0	202 (47.8%)	82 (48.2%)	120 (47.4%)	
N+	221 (52.2%)	88 (51.8%)	133 (52.6%)	0.871
Tumor differentiation				
Well	126 (29.8%)	41 (24.1%)	85 (33.6%)	
Moderate	211 (49.9%)	94 (55.3%)	117 (46.2%)	
Poor	86 (20.3%)	35 (20.6%)	51 (20.2%)	0.093
Positive margin	63 (14.9%)	28 (16.5%)	35 (13.8%)	0.455
Free flap type				
Radial forearm flap	202 (47.8%)	84 (49.4%)	118 (46.6%)	
Anterolateral thigh flap	132 (31.2%)	56 (32.9%)	76 (30.0%)	
Fibula flap	89 (21.0%)	30 (17.6%)	59 (23.3%)	0.369

* *PBT, perioperative blood transfusion*.

A radial free forearm flap operation was performed in 202 (47.8%) patients, an anterolateral thigh flap operation was performed in 132 (31.2%) patients, and a free fibula flap operation was performed in 89 (21.0%) patients (Table 1).

A total of 170 (40.2%) patients required PBT, and 100 patients received blood transfusion of 4 units. A total of 50 patients received blood transfusion of 6 units, and 20 patients received blood transfusion of 8 units. In these 170 patients, 20 (11.8%) patients received only preoperative blood transfusion, 109 (64.1%) patients received intraoperative and postoperative blood transfusion simultaneously, and 41 (24.1%) patients received only intraoperative blood transfusion.

In patients with PBT, 10 (5.9%) flaps required exploration revision, SSI occurred in 18 (10.6%) patients, and flap necrosis was noted in 6 (3.5%) patients. In patients without PBT, 18 (7.1%) flaps required exploration revision, SSI occurred in 28 (11.1%) patients, and flap necrosis was noted in 10 (4.0%) patients. None of these differences were significant (all *p* > 0.05) (Table 2).

**Table 2:** Comparison of short term outcome in patients with and without perioperative blood transfusion (PBT).

**Variables**	**PBT (*n* = 170)**	**No PBT (*n* = 253)**	***p***
Flap revision	10 (5.9%)	18 (7.1%)	0.617
Surgical site infection	18 (10.6%)	28 (11.1%)	0.877
Flap necrosis	6 (3.5%)	10 (4.0%)	0.823

**Figure 1:**
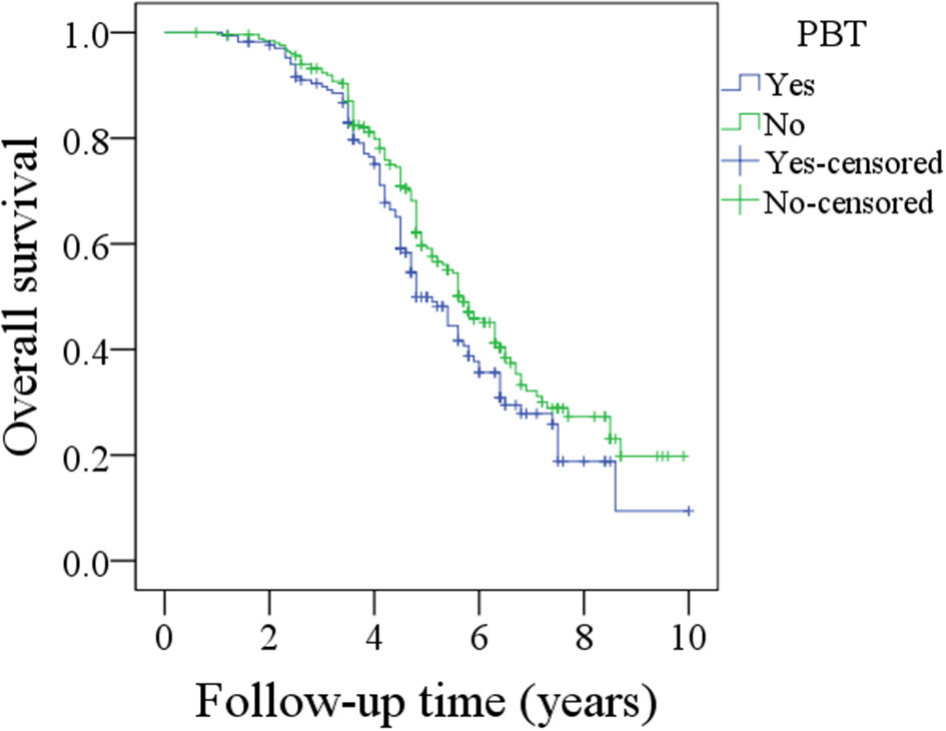
Comparison of overall survival between patients with and without perioperative blood transfusion (PBT) (*p* = 0.041).

**Figure 2:**
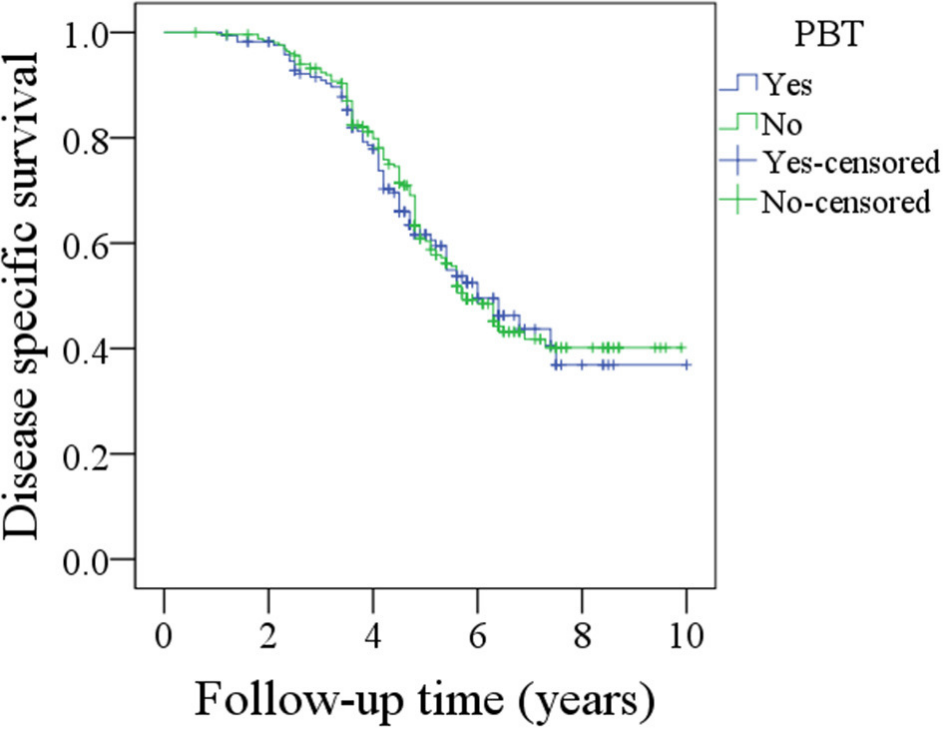
Comparison of disease specific survival between patients with and without perioperative blood transfusion (PBT) (*p* = 0.799).

During the follow-up period lasting a mean of 4.9 years, in patients with PBT, 157 patients received postoperative radiotherapy or chemoradiotherapy, and recurrence occurred in 92 patients, including 17 cases locally, 30 cases regionally, 35 cases locally and regionally, and 10 cases distantly. A total of 102 patients died, 72 patients of whom died from the cancer. The 5-year OS and DSS rates were 49 and 60%, respectively. In patients without PBT, 228 patients received postoperative radiotherapy or chemoradiotherapy, and recurrence occurred in 155 patients, including 30 cases locally, 45 cases regionally, 65 cases locally and regionally, and 15 cases distantly. A total of 140 patients died, 120 of whom patients died from the cancer. The 5-year OS and DSS rates were 59 and 60%, respectively. The OS difference between the two groups was significant (Figure 1, *p* = 0.041), but the DSS difference between the two groups was not significant (Figure 2 and Table 3, *p* = 0.799). A further Cox model analysis confirmed the significance of PBT in predicting the OS (Table 4).

**Table 3:** Univariate and cox model analyses of disease specific survival in the 423 enrolled patients.

**Variables**	**Univariate**	**Cox model**	
	**Log-rank test**	***p***	**HR[95%CI]**
Age (<40 vs ≥40)	0.336		
Sex	0.415		
Smoker	0.086		
Drinker	0.365		
Systemic disease	0.326		
Primary cancer site			
Tongue vs. others	0.004	<0.001	2.431 [1.311–6.472]
Pathologic T stage			
T4 vs. T2+T3	<0.001	<0.001	3.672 [1.478–9.443]
Tumor differentiation			
Well			
Moderate		<0.001	2.007 [1.241–5.962]
Poor	<0.001	<0.001	3.477 [2.017–9.004]
Positive margin	<0.001	<0.001	5.765 [2.200–14.778]
Pathologic N stage			
N+ vs. N0	<0.001	<0.001	3.462 [1.522–9.467]
ECOG^!^			
0			
1			
2	0.674		
Serum albumin level	0.567		
ASA status			
I			
II			
III	0.453		
PBT^*^	0.799		

! *ECOG, Eastern Cooperative Oncology Group*.

* *PBT, perioperative blood transfusion*.

**Table 4:** Univariate and Cox model analyses of overall survival in the 423 enrolled patients.

**Variables**	**Univariate**	**Cox model**	
	**Log-rank test**	***p***	**HR[95%CI]**
Age (<40 vs ≥40)	0.257		
Sex	0.684		
Smoker	0.114		
Drinker	0.285		
Systemic disease	<0.001	<0.001	2.356 [1.247–5.336]
Primary cancer site			
Tongue vs. others	0.086		
Pathologic T stage			
T4 vs. T2+T3	<0.001	<0.001	2.564 [1.114–5.875]
Tumor differentiation			
Well			
Moderate		<0.001	2.345 [1.567–6.335]
Poor	<0.001	<0.001	3.641 [2.478–7.998]
Positive margin	<0.001	<0.001	1.995 [1.256–2.337]
Pathologic N stage			
N+ vs. N0	<0.001	<0.001	3.221 [1.657–8.665]
ECOG^!^			
0			
1		<0.001	1.888 [1.087–3.276]
2	<0.001	<0.001	2.778 [1.922–5.887]
Serum albumin level	0.334		
ASA status			
I			
II		<0.001	2.654 [1.227–4.889]
III	<0.001	<0.001	3.698 [2.658–9.664]
PBT^*^	0.041	0.012	2.005 [1.087–4.003]

! *ECOG, Eastern Cooperative Oncology Group*.

* *PBT, perioperative blood transfusion*.

**Figure 3:**
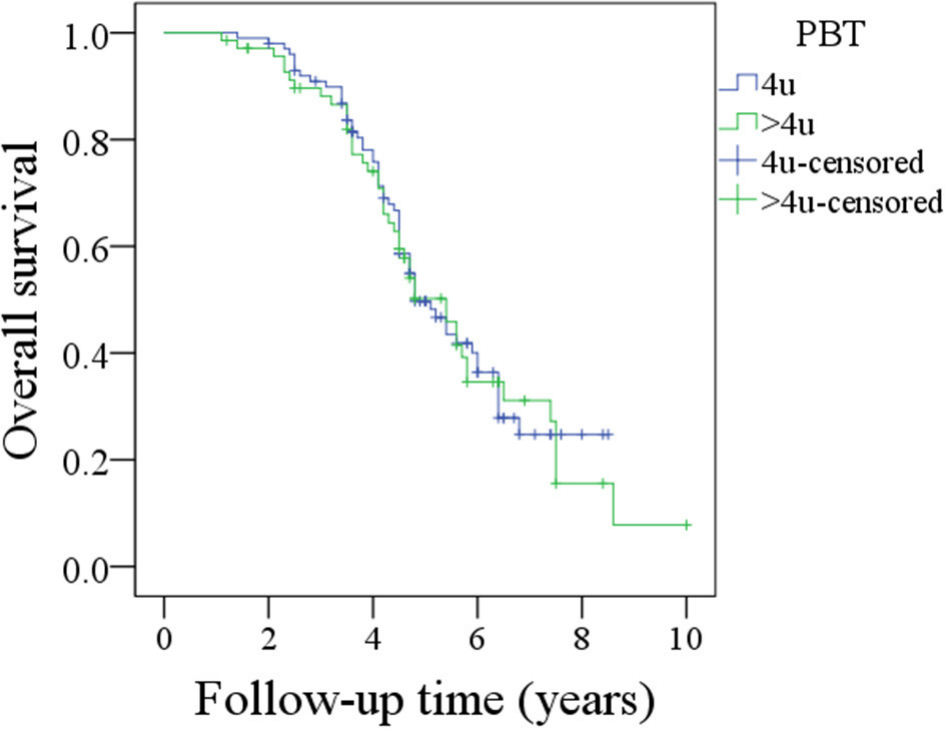
Comparison of overall survival between patients with 4 units perioperative blood transfusion (PBT) and patients with >4 units PBT (*p* = 0.839).

The association between the amount of PBT and OS was further analyzed in the 170 patients. In the 100 patients receiving 4 units of PBT, the 5-year OS rate was 50%; in the 70 patients receiving more than 4 units of PBT, the 5-year OS rate was 49%; this difference was not significant (Figure 3, *p* = 0.839).

## Discussion

The most significant finding in the current study was that PBT did not increase the risk of flap revision, SSI, flap necrosis, or cancer-caused death. However, it was associated with decreased OS in a dose-dependent manner. This provided powerful clinical value: although PBT was sometimes inevitable, a restrictive transfusion policy must be ensured.

Postoperative complications were an important aspect in perioperative management. Innutrition, including anemia, had significant negative influence on wound healing. One main goal of PBT was to improve low hemoglobin levels. It was interesting, as such, to analyze the change in the true incidence of postoperative complications after PBT. Böck et al. (19) analyzed the postoperative infection rate in 151 patients with laryngeal SCC and found 34.2% of the 111 patients with PBT developed postoperative infection. This was comparable to the 40% in 40 patients without PBT. However, in this research, local and systemic infections were evaluated together, and the blood products used included packed red cells and whole blood. von Doersten et al. (20) selected 104 patients with head and neck SCC, in whom 51 cases received PBT. Postoperative infections and fistula formations occurred in 26 and 12% patients, respectively. PBT was related to infectious complications in a univariate analysis, but not in a multivariate analysis. Sturgis et al. (21) enrolled 61 patients with head and neck cancer and noted 41% of the 34 patients with PBT had postoperative infections. This was significantly higher than the 12% in the 27 patients without PBT, but their sample size was very small, and different types of cancer were analyzed together. Recently, Danan et al. (18) assessed the outcome of PBT in 167 patients with head and neck cancer undergoing free tissue transfer. The rates of wound infection in patients who received 0, 1, 2, or 3 units blood were 13.3, 21.2, 33.3, and 31.2%, respectively. In addition, patients receiving ≥2 units had higher wound infection rates than patients receiving <2 units, but there was no difference regarding flap revision or failure between the two groups. No other studies on the association between PBT and postoperative complication in head and neck cancer were available. The current study had the largest sample size to the best of our knowledge, and our findings partially supported the conclusions of Danan et al. (18). However, we noted the PBT did not increase the SSI rate. This difference could be explained by the different distribution of ASA class: about half of the patients in the research performed by Danan et al. (18) were staged 3/4.

Oncologic survival was another important aspect of the assessment of the effect of PBT. PBT was more likely to occur in patients with high ASA score and low preoperative hemoglobin levels (22), and PBT-related immunosuppression was a more involved subject of discussion. It was characterized by increased suppressor T-cell activity and appeared to be dependent on the number of transfusions. As such, theoretically, PBT could both affect the OS and DSS. In a study conducted by Szakmany et al. on 559 patients with oral or oropharyngeal SCC (11), after adjusting for relevant prognostic factors in a Cox regression, the hazard ratio for patients receiving ≥3 units of PBT relative to those not transfused was 1.52 for cancer-caused death and 1.52 for overall mortality. However, there was no DSS difference between patients without PBT and with 1–2 units of PBT. Fenner et al. (3) enrolled 223 patients undergoing free flap reconstruction for oral SCC, in which only 3% of the patients did not receive PBT. In the analysis, the authors selected a cut-off value of 4 units for PBT, and their multivariate model revealed that patients who received no or <4 units of PBT did not tend to have a prolonged OS compared with those who received >4 units of PBT. Danan et al. (18) also analyzed the outcome in 167 patients undergoing free tissue transfer for head and neck SCC, and found that ≥3 units of PBT was associated with a nearly 3-fold increased risk of overall death and 2.5-fold increased of for disease recurrence. In addition, the PBT was the strongest prognostic factor. Baumeister et al. (23) evaluated the significance of PBT in 354 patients with surgically treated head and neck SCC, noting that preoperative anemia and PBT both significantly decreased the OS but had little effect on disease-free survival. Chau et al. (24) retrospectively reported recurrence and mortality rates were significantly different between transfusion and no-transfusion groups in 520 patients, in favor of the no-transfusion group. Taniguchi et al. (25) studied a consecutive series of 105 patients with stage II-IV oral SCC, reporting that PBT was required in 64 (61%) patients, and ≥3 units of PBT was an independent prognostic indicator. The calculated odds ratio for death after ≥3 units transfused was 5.79. Therefore, there clearly remains a certain degree of controversy with regard to the association between PBT and prognosis in the current literature. There were at least three aspects contributing to the variation. First, different objects were enrolled, and PBT was frequently required in advanced-stage patients, patients undergoing free flap reconstruction, or patients with severe anemia. Furthermore, PBT-related immunosuppression was usually induced by cells, biosoluble factors, and microchimerism, but the transfused allogeneic white blood cells were responsible for the majority of observed immunosuppression effects (26). In addition, the blood products varied across studies. Third, it was important to differentiate whether PBT was required for preoperative anemia, intraoperative blood loss, or both. In the current study, it was noted that PBT was related to decreased OS but not DSS in a dose-dependent manner. This finding was interesting, and partially explained by the fact that more patients receiving PBT had a poorer ECOG status, meaning they were more likely to die of other non-cancer-related causes. We only included patients undergoing free flap surgeries, and the blood products were leukocyte-depleted. This said, comparing the outcomes among patients who received PBT for different reasons remained difficult, especially in the context of grouping patients with moderate anemia requiring PBT. More high-quality studies were needed to clarify these questions.

Limitations of the study must be acknowledged. First, a selective bias due to the retrospective nature of the study was inevitable. Second, there were other short-term indicators that were not analyzed in current study, which could potentially result in interesting findings.

In summary, in patients with free flap reconstruction for oral SCC, PBT did not increase the short-term complication rate or cancer-linked mortality; however, it was related to elevated overall death risk.

## Data Availability Statement

The original contributions presented in the study are included in the article/supplementary Material, further inquiries can be directed to the corresponding authors.

## Ethics Statement

The studies involving human participants were reviewed and approved by Zhengzhou University institutional research committee, and all participants provided written informed consent for medical research prior to the initial treatment. All experiments were performed in accordance with the relevant guidelines and regulations. The patients/participants provided their written informed consent to participate in this study.

## Author Contributions

All authors made the contribution in study design, manuscript writing, studies selecting, data analysis, study quality evaluating, and manuscript revising. All authors have read and approved the final manuscript.

## Conflict of Interest

The authors declare that the research was conducted in the absence of any commercial or financial relationships that could be construed as a potential conflict of interest.
